# The Bionomics of the Cocoa Mealybug, *Exallomochlus hispidus* (Morrison) (Hemiptera: Pseudococcidae), on Mangosteen Fruit and Three Alternative Hosts

**DOI:** 10.3390/insects8030075

**Published:** 2017-07-25

**Authors:** Murni Indarwatmi, Dadang Dadang, Sobir Ridwani, Endang Sri Ratna

**Affiliations:** 1 Graduate student of Entomology, Faculty of Agriculture, Bogor Agricultural University, Bogor~16680, West Java, and working at the National Nuclear Energy Agency, Jakarta 12440, Indonesia; 2 Faculty of Agriculture, Bogor Agricultural University, Bogor 16680, Indonesia; dadangtea@ipb.ac.id (D.D.); rsobir@yahoo.com (S.R.)

**Keywords:** *Exallomochlus hispidus*, fecundity, insect development and reproduction, mealybug, quarantine pest

## Abstract

The cocoa mealybug, *Exallomochlus hispidus* Morrison (Hemiptera: Pseudococcidae) is known to attack mangosteen, an important fruit export commodity for Indonesia. The mealybug is polyphagous, so alternative host plants can serve as a source of nourishment. This study aimed to record the bionomics of *E. hispidus* on mangosteen (*Garcinia mangostana* L.) and three alternative hosts, kabocha squash (*Cucurbita maxima* L.), soursop (*Annona muricata*, L.), and guava (*Psidium guajava* L.). First-instar nymphs of the *E. hispidus* were reared at room temperature on mangosteen, kabocha, soursop, and guava fruits until they developed into adults and produced nymphs. Female *E. hispidus* go through three instar stages before adulthood. The species reproduces by deuterotokous parthenogenesis. *Exallomochlus hispidus* successfully developed and reproduced on all four hosts. The shortest life cycle of the mealybug occurred on kabocha (about 32.4 days) and the longest was on guava (about 38.3 days). The highest fecundity was found on kabocha (about 100 nymphs/female) and the lowest on mangosteen (about 46 nymphs/female). The shortest oviposition period was 10 days on mangosteen and the longest, 10 days, on guava. These findings could be helpful in controlling *E. hispidus* populations in orchards.

## 1. Introduction

*Exallomochlus hispidus* Morrison (Hemiptera: Pseudococcidae) is a mealybug native to Southeast Asia [1]. It is known to be a polyphagous insect, and members of 25 plant families have been recorded as hosts [2]. Williams reported that the mealybug occurs on 41 host plants belonging to 30 families in the Southeast Asian region [1]. In Indonesia, *E. hispidus* attacks high-value fruit crops such as mangosteen (*Garcinia mangostana* L.), soursop (*Annona muricata* L.), guava (*Psidium guajava* L.), rambutan (*Nephelium lappaceum* L.), durian (*Durio zibethinus* Murray), and duku/langsat (*Lansium domesticum* Correa) [1]. *Exallomochlus hispidus* has become a serious quarantine because it is often carried on the mangosteen fruit, an important export fruit commodity for Indonesia [3].

There are no reports of actual economic losses associated with any reduction in yield of *G. mangostana* fruits because of *E. hispidus* infestation. However, this mealybug can reduce the performance of the plants because its sugary honeydew excretions on nearby plant surfaces serve as a growth medium for sooty mold fungi, impairing photosynthesis and blackening the fruits [1]. The mealybug colonies are often surrounded by ants, which could put off consumers, particularly when handling or eating the fruit. The primary problem encountered with mealybugs affecting the economic value of the mangosteen fruits is the strict phytosanitary requirements of importing countries, like Australia, the United States, and New Zealand [3]; for example, New Zealand includes *E. hispidus* among the listed quarantine pests from Indonesia [4].

Farmers in Indonesia usually cultivate *G. mangostana* by intercropping with other fruit plants, like banana (*Musa paradisiaca* L.), coconut palm (*Cocos nucifera* L.), duku/langsat, durian, guava, jackfruit (*Artocarpus heterophyllus* Lam.), soursop, and rambutan, among others [5]. The intercropped plants also serve as alternate hosts for *E. hispidus*, which infest mangosteen, and could influence its life cycle parameters. Past research has shown that the host plant species can have a significant effect on mealybug development and reproduction, for example in the mango mealybug (*Rastrococcus iceryoides*) [6], and biological parameters can be changed when insects are reared on alternative media in the laboratory. For example, kabocha squash (*Cucurbita maxima* L.) is commonly used as a medium for rearing mealybugs in the laboratory [6,7,8]. Research on the bionomics of *E. hispidus* on mangosteen and other hosts has not yet been reported. Therefore, this research aimed to investigate the development and reproduction of *E. hispidus* on mangosteen and some of the other fruits that are cultivated around it: kabocha, soursop, and guava. The host plants used in this research represent some of the economically important fruit and intercropping plants grown on mangosteen plantations in Indonesia.

## 2. Materials and Methods

### 2.1. The Preparation of Fruit Hosts and Rearing of Mealybug E. hispidus

The source used for *E. hispidus* colonies was infested *G. mangostana* fruits from Bogor, West Java, Indonesia. The mealybugs were reared on kabocha fruits in the laboratory for three generations before the start of the experiment. When the size of the *E. hispidus* colony reached the maximum or the kabocha fruits began to wilt, the kabocha was replaced with fresh fruit. The colony was maintained at room conditions (27 °C ± 1 °C, in 70% ± 10% relative humidity, and a photoperiod of 12 h light and 12 h dark).

The experimental host fruits were mangosteen, guava, kabocha, and soursop. Mature mangosteen and guava fruits were bought from a supermarket, while kabocha and soursop fruits were bought at Kramat Jati Central Market, Jakarta. Before this experiment was conducted, *E. hispidus* was reared on host fruits (mangosteen, guava, kabocha, and soursop) for one generation to adapt to the experimental host [6]. The experimental host fruits were first washed in a sodium hypochlorite (3% *v*/*v*) solution three times to avoid any mold contamination of the fruit surface, followed by air-drying before being placed individually in insect rearing cages. The mealybugs were transferred using a soft brush from the adaptation to the experimental fruits [6,7]. Each rearing cage was made from a plastic cylinder (17 cm bottom diameter and 21 cm top diameter × 20 cm height) and the top was covered with a fine screen cloth to prevent the crawlers from escaping. The experimental units were maintained at room conditions as describe above. The experimental fruits were replaced twice a week and mealybugs transferred to fresh fruits using a soft brush.

### 2.2. Morphometrics of Adult Female E. hispidus

Twenty crawlers of *E. hispidus* were isolated from each adaptation host fruit and transferred into rearing cages containing experimental host fruit: mangosteen, guava, kabocha, and soursop fruit. The colonies were maintained under ambient temperatures and humid room conditions. Each adult female mealybug, 3–4 days after molting to the adult stage, was used for morphometric study and prepared as a whole-body-slide-mounted specimen observed under a stereo microscope Motic SMZ-171 using the method described in William and Watson [9]. The body length (mm) was measured digitally along the dorsal midline from the front of the head to the tip of the abdomen, and the width (mm) measured transversely between the lateral margins of the metathorax. The measurement data of 20 individuals from each host were automatically recorded using the Motic Image Plus version 2.0 (Motic Incorporation Ltd, Causeway, Hongkong, China) program [10].

### 2.3. Growth, Development, and Reproduction of E. hispidus

Twenty crawlers of *E. hispidus* were isolated from the adaptation colony and separately maintained in rearing cages on the experimental host fruit (i.e., mangosteen, guava, kabocha, and soursop fruit) as described previously. The growth and development of 20 crawlers each host were monitored until their death. Record of mealybug development periods on each host fruit was recorded every day, including nymphal stadium, prebirthing, birthing, postbirthing, and adult longevity. Molting was determined by finding white exuviae in the cages. The life cycle of the mealybug was recorded as starting with nymphs being laid, and ended with adulthood at the beginning of the birthing period. The fecundity of emerging adults was observed by counting the progeny daily until reproduction ceased (the postbirthing period).

### 2.4. Water, Nitrogen, and Total Sugar Content of the Host Fruits

Variations in the levels of nutrient such as water, nitrogen, and sugar in each host play an important role in the viability, growth, and reproduction of the mealybugs. Measurements of water, nitrogen, and total sugar content were made for the whole fruits of soursop, kabocha, and guava, and for the rind of mangosteen. The water content analysis was based on the difference between wet and dry weights. The estimation of nitrogen and total sugar contents were made using the Kjeldahl and Anthrone methods, respectively [9].

### 2.5. Data Analysis

The data were analyzed through one-way analysis of variance using the SAS program [10]. The means were separated by Tukey’s test on α = 0.05. The daily fecundity was analyzed by descriptive analysis and was recorded as a daily fecundity figure.

## 3. Results

### 3.1. The Morphology of E. hispidus

**Figure 1:**
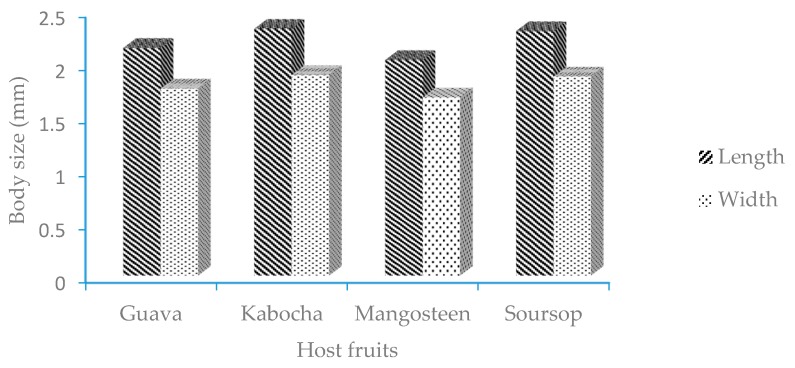
The body size of adult *E. hispidus* on mangosteen and three other host fruits.

Body length: mealybugs on the mangosteen fruit were the shortest (average length 2.03 mm) and were significantly different from the mealybugs reared on the other hosts (Figure 1). Mealybugs reared on kabocha were the longest (average length 2.33 mm), but not significantly different from those reared on soursop (average length 2.31 mm). The mealybugs reared on guava were shorter (average length 2.15 mm) than those on kabocha and soursop, but they were longer than on mangosteen (average length 2.03 mm); this difference was statistically significant. Body width: the mealybugs reared on mangosteen had the smallest width (average 1.68 mm), which was significantly different from that of mealybugs reared on the three other hosts (Figure 1). The widths of mealybugs reared on kabocha and soursop (average widths 1.89 and 1.88 mm, respectively) were larger that of those reared on mangosteen (average 1.68 mm) and guava (average 1.76 mm); these results were significantly different (Figure 1).

### 3.2. The Bionomics of E. hispidus

Female *E. hispidus* mealybugs pass through three instar nymphal stages before the adult stage. The overall duration of development of *E*. *hispidus* (from the birth of the first instar nymph to the final molt to the adult stage) varied significantly between host plants. The development of first-instar nymphs on kabocha and soursop took about 7.7 to 8.8 days, a significantly shorter time than the development on guava and mangosteen (about 8.8–9.1 days). The shortest development of the second and the third-instar nymphs on kabocha, at about 6.0 and 6.9 days, which differed significantly from the development on the three other hosts, soursop, guava, and mangosteen, (between 7.1, 7.3, 7.3 and 8.2, 8.2, 8.4 days, respectively). The shortest life cycle (about 32 days) occurred on kabocha, followed by soursop (about 35 days); the longest life cycle was on mangosteen and guava fruits (about 38 days on both hosts) (Table 1).

Starting as newly emerged adults, the female *E. hispidus* went through three periods of reproductive development before death. Table 2 shows that the prebirthing period of *E. hispidus* was about 13.5–14.0 days on mangosteen and guava host fruits, which was significantly longer than the approximately 11.8–12.0 days on kabocha and soursop. The shortest offspring production period, which is the middle stage of reproductive development, was about 6.2 days on mangosteen, similar to that on soursop (about 7.4 days) but significantly different from the longest periods, about 8.9 and 10.0 days, on kabocha and guava, respectively. At the end of reproductive development, the mealybug took a break in reproduction before dying. The postnatal period of *E. hispidus* on kabocha was about 0.6 days, which was significantly shorter than on guava (about 1.4 days) but not significantly different from that on mangosteen and guava (about 1.1–1.2 days). The longevity of adult females was not very variable between the four host fruits, although the total adult life stage on guava (about 25.3 days) was longer than on the three other hosts, mangosteen, kabocha, and soursop (about 20.7, 21.3 days).

### 3.3. The Reproduction of E. hispidus

**Figure 2:**
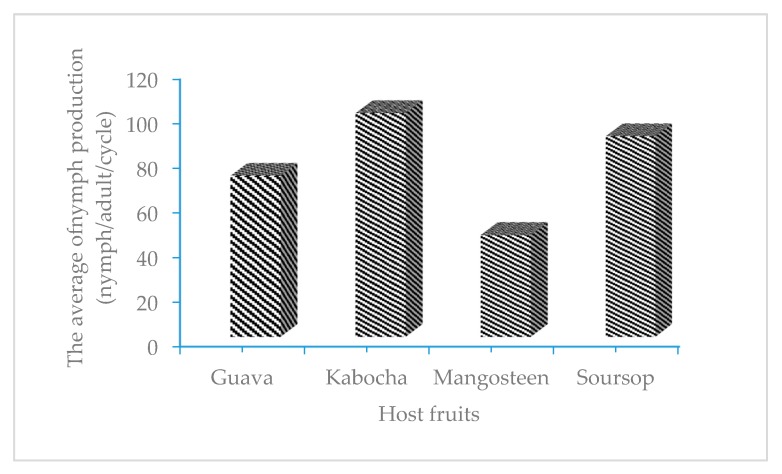
Total number of nymphs produced on mangosteen and three other host fruits.

The host plant used for rearing of *E. hispidus* mealybugs influenced the fecundity of the adults produced. Figure 2 shows that the lowest number of offspring was produced by adults fed on mangosteen followed by those reared on guava and soursop (about 33–72, 45–159, and 53–168, respectively), with averages values of 46, 72, and 90 nymphs, respectively. The highest numbers occurred on kabocha fruit, with between 72 and 203 nymphs and an average of 101 nymphs. The nymph production on mangosteen was significantly different from that on kabocha, soursop, and guava, while nymph production on kabocha was not significantly different from that on soursop but was still significantly more than that on guava fruits.

**Figure 3:**
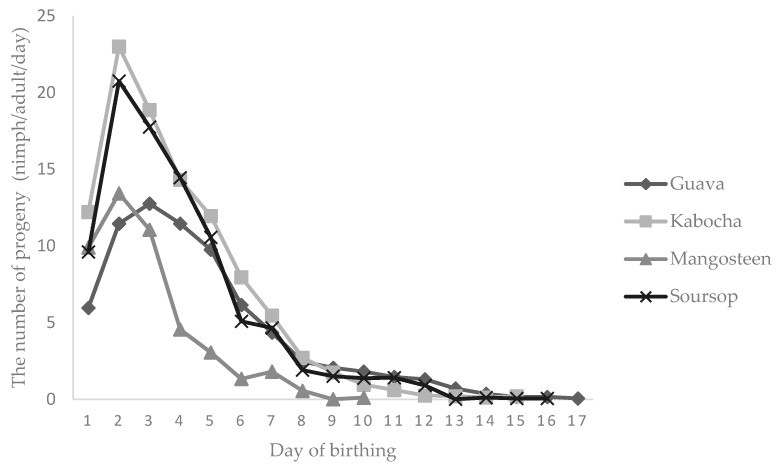
The daily fecundity of mealybug *E. hispidus* on mangosteen and three other host fruits.

The nymphal production in the early birthing period was highest and declined over the period with time of reproduction. Production of nymphs was the greatest on the second day for all host fruits, except guava, for which the highest production was on the third day. The longest birthing period was on guava (17 days), while the shortest was on mangosteen (10 days) (Figure 3).

### 3.4. Water, Nitrogen, and Total Sugar Content in Host Fruits

The nutrient content differed according to the host fruit (Table 3). Relative to the other fruits, the water content was the highest in the kabocha fruit (93.8%) and the lowest in the mangosteen fruit (64.5%) (Table 3). The nitrogen content was also the highest in the kabocha (2.0%) and the lowest in the mangosteen fruit (0.4). The total sugar content was the highest in the guava fruit (10.5%) and the lowest in the mangosteen fruit (2.0%).

## 4. Discussion

The body of adult female of *E. hispidus* is oval, fairly flat, and light tan in color. The body of the first-instar nymph (crawler) is light tan in color and is covered with a thin layer of mealy white wax that becomes thicker as the nymph grows. The first-instar nymphs (or crawlers) walk about actively to disperse to other hosts, or are picked up and transferred by the wind [11]; so crawlers can move from mangosteen plants to other host plants in the surrounding area. The second and third-instar nymphs as well as the adults are largely sessile and live in a colony.

The *E. hispidus* reared on different hosts had different adult body sizes (Figure 1). Those fed on mangosteen and guava were smaller than those reared on kabocha and soursop. Differences in body size on different hosts also have been reported for *Rastrococcus iceryoides* (Green) reared on kabocha and mango (*Mangifera indica* L.), whose bodies were longer than those reared on coffee (*Coffea arabica* L.), and their width on kabocha, mango, Jerusalem thorn (*Parkinsonia aculeata* L.), and pigeon pea (*Cajanus cajan* (L.) Millspaugh) was greater than in those on weeping fig (*Ficus benjamina* L.) and arabica coffee [6]. Similarly, the body size of *Dysmicoccus brevipes* (Cockerell) fed on pineapple [*Ananas comosus* (L.) Merr.] was found to be larger than that of specimens reared on galangal/kencur (*Kaempferia galanga* L.) [12].

The period of nymphal development influences the fitness of the mealybug [6]. The development of *E. hispidus* on kabocha was the shortest, followed by that on soursop, guava, and mangosteen, respectively. These findings agree with a study by Tanga et al. [6], which reported the development time of mealybug *R. iceryoides* to be shortest when reared on kabocha. The fitness of *E. hispidus* fitness is important because it will determine whether the mealybug is likely to survive an export journey, and potentially survive on a new host in the destination country. The U.S. Quarantine Agency has found specimens of *E. hispidus* on soursop, durian, mangosteen, duku/langsat, and rambutan from Indonesia, Malaysia, the Philippines, Singapore, Thailand, and Vietnam at U.S. ports of entry [1].

*Exallomochlus hispidus* mealybugs developed more slowly on mangosteen than on other hosts; however, mangosteen was still able to support the development and reproduction of the insect. Insects select host plants or certain parts of a plant that provide sufficient nutrition as their hosts to fulfill their needs [13]. The extended nymphal period on *D. brevipes* recorded by Bertin et al. was caused by low nutrition [7]. Tanga et al. [6] reported that the development of *R. iceryoides* takes longer on weeping fig and arabica coffee and there are fewer survivors on these two hosts. Nevertheless, both these hosts do support the development of *R. iceryoides*. The slower development is suspected to be caused by constituent compounds and physiological barriers that lead to *R. iceryoides* eating less, which slows development and decreases the number of survivors to adulthood [6]. Another study mentions that the total development period of *D. brevipes* on pineapple was 32.1 days, which is shorter than its development on galangal, which was 35.6 days [14].

In the present study, the host fruits were replaced twice a week and mealybugs were transferred to the fresh fruit. In nature, mealybugs live on host fruit that has not been picked, so this substrate differs from the picked fruit used in the laboratory. Furthermore, after picking the mangosteen rind quickly hardens during storage, and the physiological changes in the fruit can be expected to affect the growth of mealybugs. Maharani et al. 2016 reported that *Paracoccus marginatus* maintained on cut leaves of papaya (*Carica papaya* L.) produced 29.3–79.1 fewer eggs than those reared on papaya seedlings, which produced 157.5–324.6 eggs [14].

The results of the present study indicate that the duration of mealybug development depends on the hosts (Table 1). All four hosts supported the development and reproduction of the *E. hispidus* mealybug, which agrees with results reported by Williams [1].

*Exallomochlus hispidus* is an ovoviviparous insect; the embryos develop inside eggs that remain in the mother’s body until they hatch into the first nymphal instar stage and are ready to be birthed. This species also has deuterotokous parthenogenesis, a type of reproduction that produces both males and females. Female parents produced progeny that develop into female and small percentage of male, but male may or not may be able to mate [15]. In this experiment, no male progeny were produced, but in the rearing colony the female sometime produced male progeny, triggered by high population density within the colony.

The results of this study show that the quantity of nymphs produced and the length of the reproductive period depended on the host. The highest nymphal production was on kabocha and soursop, while the lowest was associated with guava (where the reproductive period was longest) and mangosteen (where the reproductive period was shortest). Tanga et al. [6] also reported that the host species had a significant effect on the growth and the reproduction of mealybug *R. iceryoides*, which helped in determining the parameters of plant growth and the duration of damage suffered by the plant. The reproduction period reported for *R. invadens* was relatively shorter, but the number of progeny was higher. This pattern meant that the mealybug was living on a suitable host [16]. Asiedu et al. [8] reported that the development, longevity, and total number of eggs laid by *Planococcus citri* were influenced by the yam variety they fed on.

The shorter development period and the higher number of nymphs produced on kabocha and soursop showed that these are good for *E. hispidus*, enabling it to produce fit individuals, but with reduced longevity. Kabocha can be stored for 2–3 months, so it was a suitable host for rearing *E. hispidus* mealybugs in the laboratory. Although soursop permitted them to grow quickly and reproduce numerous progeny, its shelf life is only about one week, which means it is unsuitable for laboratory rearing.

The nutrients contained in host plants, including water, nitrogen, and sugar, play an important role in the growth of insects. The nitrogen content in plants is correlated with their protein content [17,18], and, in insects, proteins are used for structural purposes, as enzymes, for transport and storage, as receptor molecules, and for morphogenesis. Sugar plays an important role as an energy source for insects. It is also required for the formation of the cuticle and can be converted into fat and protein [19].

For the above reason, the nutrition content of the host plant influences the development and reproduction of insects. In *E. hispidus*, nitrogen content affects the female body size, development, and reproduction in *E. hispidus*. The level of water, nitrogen, and total sugar content in mangosteen were the lowest among the fruits included in this study (Table 3). These attributes resulted in smaller body size, a longer developmental period, and lower nymphal production in the mealybugs. The water and nitrogen content was highest in kabocha. In both kabocha and soursop, the body size was larger, the developmental period was shorter, and the nymphal production was higher, even though the total sugar was lower. The total sugar content in guava was the highest, but the nitrogen content was lower, resulting in a smaller body size, longer developmental period, and lower nymphal production.

The results showed that the nitrogen content had a greater effect on the development and reproduction of *E. hispidus* than water and sugar content. Hogendrop et al. [19] studied how nitrogen affects the growth and the reproduction of citrus mealybug, *P. citri* on coleus [*Solenostemon scutellarioides* (L.) Codd]. Their results showed that high nitrogen led to a larger female body, shorter developmental period, and higher reproduction capacity. Similar results were reported for the aphid *Macrosiphum euphorbiae* (Thomas) (Hemiptera: Aphididae) when reared on a plant without fertilizer or with a low nitrogen level, which made the developmental period longer and the reproductive capacity lower [20]. Adult females need more protein than males for egg production [20]. Our results can be used to assess the impact of the fitness and developmental success of the *E. hispidus* population on host fruits, and this information can be used to control the mealybug in the cultivation of important fruits in Indonesia.

## 5. Conclusions

The body size, developmental period, and reproductive capacity of *E. hispidus* were influenced by the fruit host species. The lowest development time and nymphal production of *E. hispidus* were on mangosteen and the highest were on kabocha. High nitrogen levels in the fruit host made the body size larger, the developmental period shorter, and the nymphal production higher.

## Figures and Tables

**Table 1:** Durations of the immature development stages and the life cycles of *E. hispidus*.

	Average Number of Days per Developmental Stage (Days ± SE)			
	First Instar	Second Instar	Third Instar	Life Cycle
Host fruit				
Guava	8.8 ± 0.7 a	7.3 ± 0.4 a	8.2 ± 0.4 a	38.3 ± 1.8 a
Kabocha	7.7 ± 0.5 b	6.0 ± 0.6 b	6.9 ± 0.5 b	32.4 ± 1.3 c
Mangosteen	9.1 ± 0.7 a	7.3 ± 0.5 a	8.4 ± 0.7 a	38.2 ± 2.1 a
Soursop	8.0 ± 0.7 b	7.1 ± 0.5 a	8.2 ± 0.8 a	35.2 ± 2.5 b
*F*	0.0001	0.0001	0.0001	0.0001
*df*	3,76	3,76	3,76	3,76
*p*	20.60	29.89	27.36	40.80

Means within a column followed by the same letter do not differ significantly by Tukey’s tests (α = 0.05).

**Table 2:** Duration of the reproductive periods and adult longevity of *E. hispidus*.

	Period (In Days)			
	Prebirthing	Birthing	Postbirthing	Adult longevity
Host				
Guava	14.1 ± 0.8 a	10.1 ± 3.4 a	1.2 ± 0.8 ab	25.3 ± 3.0 a
Kabocha	11.8 ± 0.7 b	8.9 ± 2.7 ab	0.6 ± 0.7 b	21.3 ± 2.2 b
Mangosteen	13.5 ± 1.1 a	6.2 ± 1.6 c	1.1 ± 0.9 ab	20.7 ± 1.7 b
Soursop	12.0 ± 1.17 b	7.4 ± 2.9 bc	1.4 ± 0.8 a	20.8 ± 2.5 b
*F*	0.0001	0.0001	0.0146	0.0001
*df*	3,76	3,76	3,76	3,76
*p*	26.56	8.61	3.74	16.97

Means within a column followed by the same letter do not differ significantly by Tukey’s test (α = 0.05). The data for birthing were log transformed. The data for postbirthing were square root transformed.

**Table 3:** Water, nitrogen, and total sugar content of mangosteen and three other host fruits analyzed.

Host Fruits	Water Content (%)	Nitrogen Content (%)	Total Sugar Content (%)
Guava	88.84	0.67	10.53
Kabocha	93.77	1.97	5.65
Mangosteen	64.52	0.41	1.98
Soursop	83.09	1.06	3.11
